# Beneficial Effects of High Intensity Interval Training and/or Linseed Oil Supplementation to Limit Obesity-Induced Oxidative Stress in High Fat Diet-Fed Rats

**DOI:** 10.3390/nu13103531

**Published:** 2021-10-09

**Authors:** Carole Groussard, Claire Plissonneau, Laurie Josset, Fréderic Capel, Mathilde Mura, Etienne Gouraud, Guillaume Mairesse, Guillaume Chesneau, Nicolas Barnich, Vincent Pialoux, Nathalie Boisseau

**Affiliations:** 1 Laboratoire M2S-EA7470, Univ-Rennes, F-35000 Rennes, France; 2 Laboratoire des Adaptations Métaboliques à l’Exercice en Conditions Physiologiques et Pathologiques (AME2P), CRNH Auvergne, Université Clermont Auvergne, F-63000 Clermont-Ferrand, France; claire.plissonneau@uca.fr (C.P.); nathalie.boisseau@uca.fr (N.B.); 3 CRNH Auvergne, Microbes, Intestin, Inflammation et Susceptibilité de l’Hôte (M2iSH), USC-INRAE 2018, UMR 1071 Inserm/Université d’Auvergne, Université Clermont Auvergne, F-63000 Clermont-Ferrand, France; nicolas.barnich@uca.fr; 4 Team “Atherosclerosis, Thrombosis and Physical Activity”, Laboratoire Interuniversitaire de Biologie de la Motricité (LIBM)-EA 7424, Université Lyon 1, F-69100 Lyon, France; laurie.josset@etu.univ-lyon1.fr (L.J.); mathilde.mura@univ-lyon1.fr (M.M.); etienne.gouraud@univ-lyon1.fr (E.G.); vincent.pialoux@univ-lyon1.fr (V.P.); 5 Unité de Nutrition Humaine (UNH), CRNH Auvergne, Université Clermont Auvergne, INRAE, F-63000 Clermont-Ferrand, France; frederic.capel@inrae.fr; 6 Valorex, La Messayais, F-35210 Combourtillé, France; g.mairesse@valorex.com (G.M.); g.chesneau@valorex.com (G.C.); 7 Institut Universitaire de France, F-75231 Paris, France

**Keywords:** linseed oil supplementation, high intensity interval training, high fat diet, obesity, oxidative stress

## Abstract

High-intensity interval training (HIIT) and linseed oil (LO) supplementation are effective strategies to reduce obesity-induced oxidative stress. Our aim was to determine whether the HIIT + LO combination prevents obesity-induced oxidative stress in high fat diet (HFD)-fed rats. HFD-fed 8-week-old, male, Wistar rats were subdivided in four groups: HFD, LO (2% of sunflower oil replaced with 2% of LO in the HFD), HIIT (4 days/week for 12 weeks), and HIIT + LO. Wistar rats fed a low-fat diet (LFD) were used as controls. Epididymal and subcutaneous adipose tissue, gastrocnemius muscle, liver, and plasma samples were collected to measure oxidative stress markers (AOPP, oxLDL), antioxidant (SOD, CAT, and GPx activities) and pro-oxidant (NOx and XO) enzyme activities. Compared with the LFD, the HFD altered the pro/antioxidant status in different tissues (increase of AOPP, oxLDL, SOD and catalase activities in plasma, and SOD activity increase in liver and decrease in adipose tissues) but not in gastrocnemius. LO upregulated CAT activity and decreased NOx in liver. HIIT alleviated HFD negative effects in liver by reducing SOD and NOx activities. Moreover, the HIIT + LO combination potentiated SOD activity upregulation in subcutaneous tissue. HIIT and LO supplementation have independent beneficial effects on the pro/antioxidant balance. Their association promotes SOD activity in subcutaneous adipose tissue.

## 1. Introduction

Obesity is a complex medical condition characterized by excess body fat, leading to impaired health and increased mortality [1]. Obesity is growing worldwide, in developed and also in developing countries. Its determinants are complex and of multifactorial origin [2]. Unhealthy dietary habits (i.e., high intake of energy-dense foods and low intake of food rich in micronutrients and bioactive compounds) [3] and physical inactivity/sedentary lifestyle [4] are the main causes of obesity. In the last few decades, the Western diet (characterized by high intake of processed foods, red meat, high-fat dairy products, high-sugar foods, and pre-packaged foods) has been a significant contributor to the growing rate of obesity that increases the risk of chronic diseases [5].

The excessive fat accumulation in adipose tissue increases reactive oxygen species (ROS) production through different mechanisms and subsequently promotes systemic oxidative stress (OS) [6,7] and cardiovascular complications [8,9]. OS occurs also in other tissues, such as skeletal muscle and liver, where ectopic fat accumulation promotes ROS production [10,11]. Moreover, recent studies showed that OS is not only a consequence of obesity, but could also trigger its onset and development. Indeed, ROS play a direct role in adipogenesis (by stimulating the deposition of adipose tissue, including preadipocyte proliferation, adipocyte differentiation and growth) [12], and OS modulates all factors involved in obesity, including genetics, sleep, gut microbiome, insulin, ghrelin, inflammation, adipokines, leptin, stress, and the hypothalamic-pituitary-adrenal axis [13]. Thus, any strategy to reduce OS should have beneficial effects on obesity onset and also on the adverse consequences associated with obesity-induced OS [14].

Strategies to lower obesity-induced OS include physical activity programs (indirectly via weight loss and directly by increasing antioxidant enzyme activities), and antioxidant-rich diets [15]. A recent training modality, called “high intensity interval training” (HIIT), has proved its effectiveness for obesity management compared with the traditional “moderate intensity continuous training” (MICT) [16]. HIIT combines intense exercise of short duration intercalated with short aerobic recovery periods, and due to the reduced exercise duration and volume, it is more suitable for our modern lifestyle [17,18,19]. HIIT could exert more significant beneficial effects on abdominal and visceral fat mass (FM) in patients with overweight and obesity compared with MICT [8]. Moreover, we recently demonstrated that this training mode significantly reduces obesity-induced muscle and plasma OS markers in Zucker rats (*fa*/*fa*), a genetic model of obesity [20]. 

Concerning nutritional strategies to improve obesity-induced OS, linseed oil (LO) could be of interest. A recent systematic review of randomized controlled trials in patients with metabolic syndrome and related disorders demonstrated that LO supplementation significantly reduces malonedialdehyde (MDA), a lipid peroxidation marker, and increases the total antioxidant capacity [21]. Among the different bioactive compounds contained in LO, alpha-linoleic acid (ALA; the most abundant) [22,23], phytosterols [24], antioxidant vitamins (especially γ-tocopherol and β-carotene; present at lower concentration), and phenolic compounds [25] have a strong antioxidant activity. Moreover, to counteract the deleterious effects of the Western diet, dietary oils with modified fatty acid profiles have been manufactured to improve fatty acid intake and limit the risk of chronic diseases [26]. LO inclusion in human diets improves the fatty acid profile [27].

The specific effects of HIIT and LO supplementation on the pro/antioxidant status in obesity are well established. However, to the best of our knowledge, no study has tested their effects when combined. As previous studies showed that in some cases, the intake of antioxidant compounds can potentiate some of the adaptations induced by training [28,29], the aim of this study was to evaluate the effects of a 12-week intervention program that included physical activity (HIIT), associated or not with LO supplementation (to equilibrate the ratio of n-6/n-3 polyunsaturated fatty acids, PUFAs), on the pro/antioxidant status. This was evaluated by measuring: (a) the three main antioxidant enzymes: superoxide dismutase (SOD), catalase (CAT), and glutathione peroxidase (GPx) that scavenge ROS, particularly superoxide and hydrogen peroxide; (b) the two main prooxidant enzymes: NAPDH oxidase (NOx) and xanthine oxidase (XO) that produce superoxide anions; and (c) oxidative stress markers on lipids (MDA and oxidized low-density lipoprotein, oxLDL) and on proteins (advanced oxidation protein products (AOPP) in different tissues (epididymal and subcutaneous adipose tissues, muscle and plasma) in high fat diet (HFD)-fed Wistar rats. We hypothesized that the physical activity program (HIIT) or LO supplementation would have beneficial effects on the pro/antioxidant status and that their combination might potentiate their effects to prevent obesity-induced oxidative stress in this rat model of obesity. 

## 2. Materials and Methods

This study is a continuation of the work we previously published in *Nutrients* [30]. In the previous study, we determined whether HIIT and/or LO supplementation (to equilibrate the n-6/n-3 PUFA ratio) might prevent obesity disorders (i.e., body composition and metabolic disturbances), particularly by modulating the mucosa-associated microbiota. This second publication focuses on obesity induced-oxidative stress prevention using these two interventions (HIIT and/or LO supplementation).

### 2.1. Ethical Approval

The experimental protocol was approved by the local ethics committee (C2EA-02, Auvergne, France; APAFIS 16090-2018071208306750) and was in accordance with the current legislation on animal experimentation (European Directive 2010/63/EU on the protection of vertebrate animals used for experimental and scientific purposes). Moreover, the experiments were carried out according to the local animal welfare committee regulations.

### 2.2. Animal Model and Experimental Groups

Sixty (8-week-old) male Wistar rats were purchased from Charles River Laboratory (France). Animals were individually housed under standard conditions: constant temperature (22 ± 2 °C), free access to food and water, and reversed 12-h light/dark cycle. 

Rats were fed either a low fat diet (LFD: *n* = 12; 11.5% of fat, 19.8% of protein and 68.7% of CHO) or an in-house HFD (*n* = 48; 43.3% of fat to induce obesity, including 4% of sunflower oil, 17.3% of protein and 39.4% of CHO) ad libitum prepared by INRAE (Jouy-en-Josas, France). Each HFD diet includes 4% of AIN93M Mineral mix and 1% of AIN93Vx Vitamin mix (Supplementary file).

**Figure 1:**
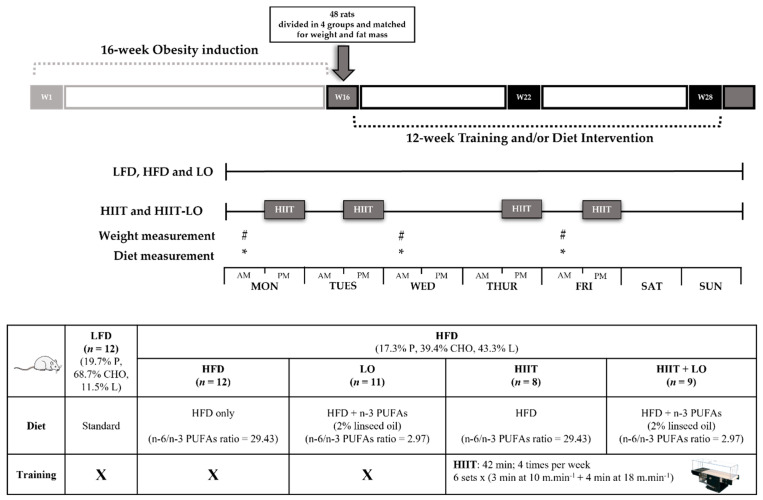
Experimental design of the study. Phase 1: rats were randomly assigned in two groups: LFD (*n* = 12) and HFD (*n* = 48) for 16 weeks. Phase 2: rats of the HFD group were divided in four groups (*n* = 12/group) matched for weight and fat mass: HFD, LO, HIIT, and HIIT + LO for 12 weeks. LFD: low-fat diet, HFD: high-fat diet, LO: linseed oil, HIIT: high-intensity interval training, PUFAs: polyunsaturated fatty acids, P: proteins, CHO: carbohydrates, L: lipids, W: weeks, #: weight measurement, *: Diet measurement.

After 16 weeks, rats in the HFD group were randomly assigned to four groups matched for body weight and total FM: (i) HFD group (*n* = 12) without intervention (control), (ii) HIIT group (n = 12) that performed the exercise program, (iii) LO group (*n* = 12) that received LO supplementation, (iv) HIIT + LO group (*n* = 12) that performed the exercise program and received LO supplementation. The four groups continued to receive the same HFD as during phase 1 (Figure 1).

Body weight and the amount of food consumed by rats were recorded 3 days per week. Body composition was measured by MRI (Echo Medical Systems, Houston, TX, USA), and epididymal fat pads were weighed post mortem. 

After 12 weeks and 48 h after the last exercise session, animals were euthanized by cervical dislocation before tissue harvesting. Whole blood samples were immediately centrifuged at 2000× *g* at 4 °C for 10 min to obtain plasma. Aliquots were stored at −80 °C until analysis. Gastrocnemius, liver and subcutaneous/epididymal adipose tissue samples were collected, weighed and immediately frozen in liquid nitrogen and stored at −80 °C until analyses. 

### 2.3. LO Supplementation

The diet in the LO groups (LO and HIIT + LO) had the same composition as the HFD, but 2% of sunflower oil was replaced by 2% of LO. The composition of all diets was analyzed by gas chromatography to confirm their n-6/n-3 PUFA ratio, and are reported elsewhere [30].

### 2.4. Training Protocol

Two weeks before the training program initiation, rats in the HIIT (*n* = 12) and HIIT + LO (*n* = 12) groups were familiarized with the treadmill using a low intensity running protocol, as described previously [20]. The treadmill habituation protocol finished three days before the beginning of the experimental period to avoid acute interference with the baseline measurements. During this familiarization period, rats that refused to run spontaneously after the treadmill started (“no runners”) were excluded from the study. 

For the training protocol (12 weeks), as described previously [20,30], animals ran on a treadmill especially designed for rats (Matsport, Clyon, France) and all sessions were performed during the dark cycle (active period).

Training was performed in a specific room at the same time in the afternoon. Animals underwent training always in the same order and on the same lane of the treadmill. Moreover, all animals, even those not training, were moved to the experimental room when other rats were training. All rats were present during all training sessions that lasted approximately two hours (four times per week).

Before each training session, animals in the HIIT groups performed a regular warm-up exercise at 10 m·min^−1^ for 5 min, and then alternated 3 min at 10 m/min and 4 min at 18 m/min (6 sets; 4 times per week for 12 weeks; for a total of 42 min/training) (Figure 1). 

Animals in the LFD and LO groups were managed identically as those in the HIIT groups, but without exercise, and were placed in the same room during the training sessions to account for the potential stress induced by environment changes.

Animals of the five groups had similar weights, body fat, and fasting glucose levels at baseline [30].

### 2.5. Biochemical Analyses

Adipose tissue gastrocnemius and liver samples were ground in liquid nitrogen, homogenized (10%, *w*/*v*) in 1X PBS/0.5 mM Ethylene Diamine Tetra Acetic Acid (EDTA) on ice, and centrifuged at 12,000× *g* at 4 °C for 10 min. Homogenates were stored in aliquots at −80 °C. Total protein concentration was determined using the BCA Protein Assays Kit (Sigma, St. Louis, MO, USA) following the manufacturer’s instructions. All the products used for oxidative stress, antioxidant and pro-oxidant marker measurements were from Sigma-Aldrich (Sigma, St. Louis, MO, USA), and spectrophotometric measurements were performed on a TECAN Infinite 2000 plate reader (Tecan, Männedorf, Switzerland). Results obtained for the adipose tissue and skeletal muscle samples were normalized to the total protein content to account for body weight variations during the experiment. Measurements were done in duplicates.

#### 2.5.1. Oxidative Stress Markers

AOPP were determined according to the method by [31] using a spectrophotometer and calibrated with a chloramine-T solution that absorbs at 340 nm in the presence of potassium iodide. The absorbance was read at 340 nm. AOPP concentrations were expressed as μmol·g^−1^ of proteins. The intra-assay coefficient of variation (CV) was 1.7%. 

The plasma concentration of MDA, as thiobarbituric acid reactive substances, was determined by extracting the pink chromogen with n-butanol and measuring its absorbance at 532 nm by spectrophotometry using 1,1,3,3-tetraethoxypropan as standard, according to the method described by Ohkawa et al. [32]. The intra-assay CV was 1.6%. 

OxLDL concentration was measured in plasma using the ELISA kit from Elabscience^®^ (Elabscience, Houston, TX, USA), according to the manufacturers’ recommendations. Absorbance was read at 450 nm.

#### 2.5.2. Antioxidant System Markers

Plasma SOD activity was determined using the method by [33], based on the degree of SOD inhibition of the reaction between superoxide radicals, produced upon hypoxanthine oxidation by xanthine oxidase, and nitro blue tetrazolium (NTB). The formed blue formazan product was read at 560 nm for 5 min. SOD activity was expressed as μmol·min^−1^·g^−1^ of proteins. The intra-assay CV was 6.7%. 

Plasma GPx activity was determined using a modified version of the method described by [34]. GPx activity is represented by the rate of nicotinamide adenine dinucleotide phosphate (NADPH) oxidation to NADP+ after addition of glutathione reductase, reduced glutathione and NADPH using H_2_O_2_ as substrate. NADPH extinction was read at 340 nm for 5 min. GPx activity was expressed as μmol·min^−1^·g^−1^ of proteins. The intra-assay CV was 3.9%. 

Plasma CAT activity was determined using the method described by [35] with H_2_O_2_ as substrate, and formaldehyde as standard. Catalase activity was determined by monitoring formaldehyde formation rate (read at 540 nm for 20 min) induced by the reaction of methanol and H_2_O_2_ using catalase as enzyme. SOD activity was expressed as μmol·min^−1^·g^−1^ of proteins. The intra-assay CV was 4.1%. 

#### 2.5.3. Pro-Oxidant Enzymes

NOx and XO activities were determined in plasma, as previously described [36] by the reaction of NTB with superoxide produced by hypoxanthine or NADPH with XO and NOx, respectively. NOx and XO activities were calculated by measuring spectrophotometrically the kinetic of appearance of the complex formed by superoxide and NTB at 560 nm for 10 min. NOx and XO activities were expressed as μmol·min^−1^·g^−1^ of proteins and the intra-assay CV were 3.6% and 3.5%, respectively.

### 2.6. Statistical Analysis

Results are expressed as the mean ± standard deviation (SD). Statistical analyses were performed with the Statistica software (version 12) (Statsoft, Tulsa, OK, USA) and figures were prepared with GraphPad Prism 7.00. Normality was checked using the Kolmogorov–Smirnov’s test. The assumption of homogeneity of variance was assessed using the Bartlett F-test. In the absence of normal distribution or variance homoscedasticity, data were log-transformed before analysis. At the end of phase 2 (training and/or LO supplementation), the *t*-test was used to compare the two experimental groups (LFD vs. HFD) to determine any HFD effect. Moreover, a one-way ANOVA (with or without repeated measures) with the four experimental groups (HIIT, HIIT + LO, LO, HFD) was performed using the Newman– Keuls post hoc test to determine group effects. Moreover, a 2-way ANOVA was used to determine the main effect of exercise (HIIT), LO supplementation, and their interaction, on the HFD [37]. Spearman correlations were used to test relationships between variables. *p* values < 0.05 were considered statistically significant.

## 3. Results

Data concerning LO supplementation effects on blood n-3 PUFAs and body composition are described in a previous study [30] that assessed HIIT and/or LO supplementation contribution to obesity prevention and the potential impact of mucosa-associated microbiota in these adaptations. As reported, LO supplementation significantly increased the content of the n-3 PUFAs eicosapentaenoic acid and docosahexaenoic acid in blood, confirming its efficiency. 

In this previous study [30], weight was monitored during the 16 weeks of phase 1. Although the cumulative food intake (kcal) did not significantly differ between groups (HFD and LFD) during this phase, weight became significantly higher in the HFD group from week 14. In addition, at the end of phase 1 (week 16), total FM, but not fat-free mass, was significantly higher in the HFD than LFD group (75.0 ± 20.6 and 56.6 ± 14.0 g; *p* < 0.05). At the end of phase 2, body weight gain was much smaller in the HIIT and HIIT + LO groups than in the HFD and LO groups due to a lower FM gain in these two actively exercising groups (*p* < 0.001). 

### 3.1. HFD Alters Plasma and Liver Pro/Antioxidant Status

**Figure 2:**
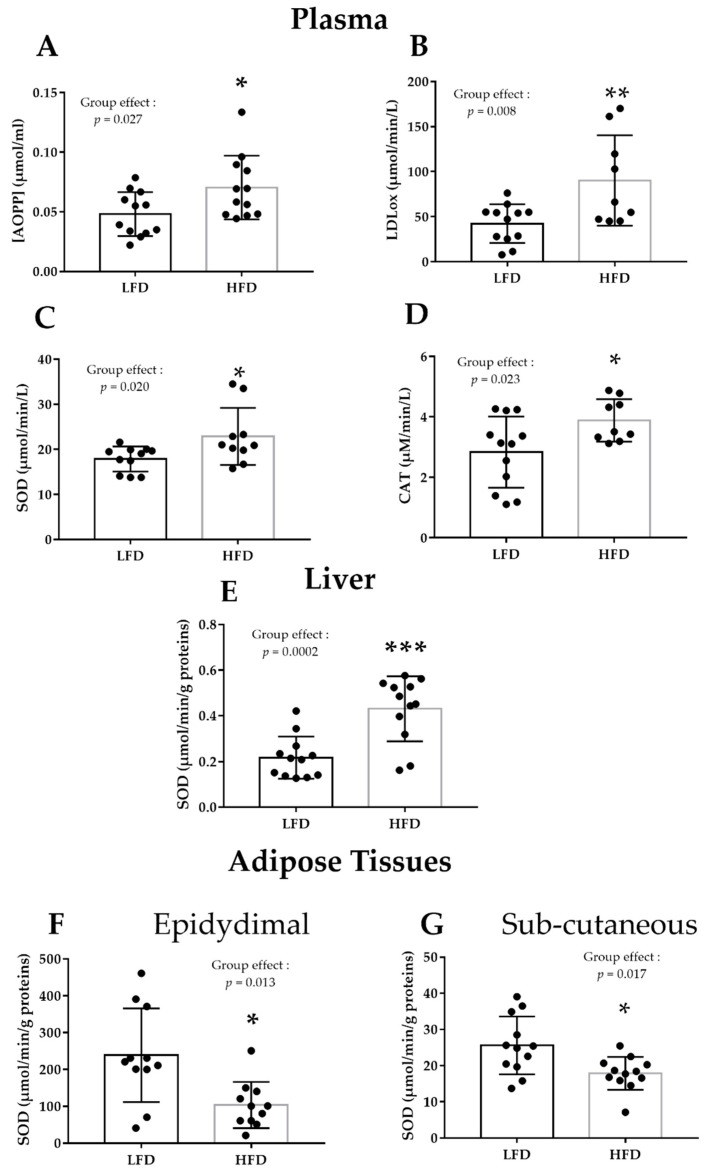
Pro/antioxidant markers in the LFD (*n* = 12) and HFD (*n* = 12) groups at the end of phase 2 (obesity induction with the HFD for 16 weeks). (**A**) Plasma AOPP concentration (µmol·mL^−1^), (**B**) plasma oxLDL activity (µmol·^−1^min·L^−1^), (**C**) plasma SOD activity (µmol·^−1^min·L^−1^), (**D**) plasma CAT activity (µmol·^−1^min·L^−1^), (**E**) liver SOD activity (µmol·^−1^min·L^−1^), and SOD activity (µmol·^−1^min·L^−1^) in (**F**) epididymal (**G**) and subcutaneous adipose tissue in the LFD and HFD groups. Data are the mean ± SD. * *p* < 0.05, ** *p* < 0.01, *** *p* < 0.001, compared with LFD (*t*-test). •: indicates the measured values of the individual rats. AOPP: advanced oxidation protein products, CAT: catalase, HFD: high fat diet, HIIT: high-intensity interval training, LFD: low fat diet, oxLDL: oxidized low-density lipoprotein, SOD: superoxide dismutase.

Plasma pro/antioxidant status was significantly altered by the HFD (Table 1, Figure 2A–D). After 16 weeks of HFD, AOPP and oxLDL (oxidative stress markers) were increased (+46%, *p* < 0.05, and +114%, *p* < 0.01), respectively) (Figure 2A,B), as well as SOD and catalase activities (antioxidant enzymes; +40% and +35%, respectively; *p* < 0.05) (Figure 2C,D). In the HFD group, SOD activity increased also in liver (+99%, *p* < 0.001) (Figure 2E), whereas it decreased in epidydimal and subcutaneous adipose tissue samples (−56% and −30%, respectively; *p* < 0.01) (Figure 2F,G). No HFD effect was observed in gastrocnemius (Table 1).

### 3.2. LO Supplementation Exerts Beneficial Effects in Liver by Upregulating CAT Activity and by Decreasing NOx Activity

**Figure 3:**
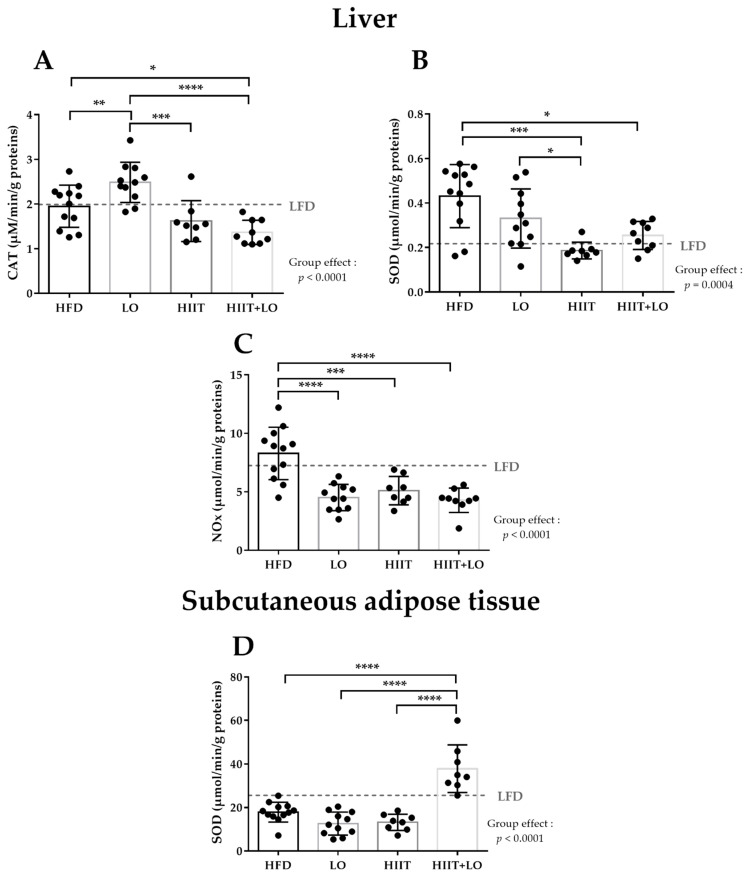
CAT (**A**), NOx (**B**), and SOD activities (**C**) in liver and SOD activity in subcutaneous adipose tissue (**D**) (µmol^−1^·min·L^−1^) in the HFD (*n* = 12), LO (*n* = 11), HIIT (*n* = 8), and HIIT + LO (*n* = 9) groups at the end of phase 2 (training and/or LO supplementation for 12 weeks). Data are the mean ± SD. * *p* < 0.05, ** *p* < 0.01, *** *p* < 0.001, **** *p* < 0.0001 (one-way ANOVA). •: indicates the measured values of the individual rats. CAT: catalase, NOx: NADPH oxidase, SOD: superoxide dismutase, LFD: level observed in rats fed the low fat diet.

LO supplementation significantly increased CAT activity in liver by 27% (*p* < 0.01) and decreased NOx activity by 45% (*p* < 0.001) (Figure 3A,C). LO supplementation did not have any effect on the other pro/antioxidant markers in liver and also in plasma, gastrocnemius and adipose tissues (Table 2). 

### 3.3. HIIT Alleviates HFD Negative Effects by Reducing SOD and NOx Activities in Liver

HIIT significantly decreased SOD activity in liver (−57% compared with the HFD group) to a similar level as in the LFD group (0.217 ± 0.092 in the LFD group and 0.186 ± 0.037 in the HIIT group) (Figure 3B). HIIT also significantly reduced NOx activity in liver (−38%; *p* < 0.01). 

HIIT did not have any effect on the other pro/antioxidant markers in liver and muscle and adipose tissue (Table 2). 

### 3.4. The HIIT and LO Combination Potentiates Their Effect on SOD Activity in Subcutaneous Adipose Tissue

HIIT and LO supplementation on their own did not alter SOD activity in the subcutaneous adipose tissue, but they had a positive synergistic effect when combined (+112%) (Figure 3D). 

The HIIT and LO combination did not potentiate their single actions in liver, although on their own they showed significant specific effects: HIIT decreased NOx and SOD activities, and LO supplementation decreased NOx activity (Figure 3B,C).

No effect of HIIT and/or LO combination was observed in plasma and epidydimal adipose tissue (Table 2). 

## 4. Discussion

This study is the first to compare the isolated and combined effects of two interventions (HIIT and LO supplementation) on the pro/antioxidant status of different tissues in HFD-fed Wistar rats. We hypothesized that HIIT or LO supplementation alone would have beneficial effects on the pro/antioxidant status and that their combination might potentiate these effects. As expected, HIIT and LO supplementation had independent beneficial effects on the pro/antioxidant balance in different tissues. HIIT alleviated HFD negative effects in liver by reducing SOD and NOx activities, and LO supplementation induced beneficial effects in liver by upregulating CAT activity and by decreasing NOx activity. The HIIT and LO combination potentiated their positive effect on SOD activity in the subcutaneous adipose tissue.

We chose a model of obesity induced by a Western-style diet, rather than a genetic model (e.g., Zucker rats) to better reflect the current causes of the global obesity epidemic. Indeed, despite the multifactorial etiology of obesity, its rate and incidence are increasing, suggesting that environmental and behavioral factors, particularly dietary factors, are the major contributors of obesity [38]. As expected, besides a significant increase of body weight and FM [30], the HFD increased systemic OS, as shown by the quantification of different pro/antioxidant markers in plasma [39,40,41]. Moreover, as previously shown, the HFD modified, but to a lesser extent, the pro/antioxidant status also in liver and adipose tissue [42,43].

LO has many beneficial effects on health, attributed to its composition. LO contains ALA (ω-3:18-3), linoleic acid (ω-6:18-2), and oleic acid (ω-9:18-1) as well as many other active and useful compounds, particularly phytochemical (phenolic acids and lignans) and tocopherols. Its antidiabetic, anti-inflammatory, anti-cancer, and cardioprotective actions are mainly attributed to ALA [44,45,46,47]). LO also contains phytosterols [24], antioxidant vitamins (especially γ-tocopherol and β-carotene, at lower concentration), lignans [48], and phenolic compounds [25] that contribute to its antioxidant properties, in addition to ALA [49]. Moreover, we previously showed that compared with sunflower oil, LO contains higher levels of n-3 PUFA and has a better n-6/n-3 PUFA ratio [30]. Therefore, LO can be an efficient and cheaper strategy to rebalance the n-6/n-3 PUFA ratio (instead of fish that is more expensive [50].

In line with previous findings, we demonstrated that LO could upregulate the antioxidant enzyme CAT in liver, although in our study HFD did not affect this marker. This result is important because antioxidant enzymes constitute the cell defense system that plays a major role in lowering oxidative stress by scavenging ROS. Han et al. [51] demonstrated in Western-type diet-fed mice that LO consumption reduces liver ROS and serum and liver MDA and also increases antioxidant enzymes (e.g., glutathione level and SOD activity) in serum and liver. Furthermore, in rats with fructose-induced metabolic syndrome, LO protects against protein and lipid damage [52] by increasing the blood enzymatic antioxidant defenses. LO might increase liver CAT through upregulation of the gene encoding this enzyme, as demonstrated by Jangale et al. [53] in a rat model of type 2 diabetes, and by decreasing enzyme glycation and the formation of advanced glycation end products due to lower glycemia [54]. 

To our knowledge, we are the first to demonstrate the effect of LO supplementation on NOx activity. This multi-component complex is a key ROS producer in many cell types, including liver cells, by catalyzing reactions from molecular oxygen to superoxide anion. By lowering its activity in liver and consequently ROS production, LO supplementation exerted beneficial effects in HFD-fed rats. El Midaoui et al. [55] reported similar results in the aorta of glucose-fed rats after 5 weeks of argan oil supplementation, also rich in tocopherols. Concerning the molecular mechanisms involved in the NOx activity decrease, Shen et al. [56] demonstrated in a rat model of atherosclerosis that omega-3 fatty acids attenuate NOx activity by decreasing the mRNA and protein expression of the NOX subunits p47phox, gp91phox, and p67phox. Moreover, Richard et al. [57] showed that in human aortic endothelial cells, the omega-3 fatty acid docosahexaenoic acid decreases NOx4 expression and activity via group V phospholipases A2 and that these effects are mediated by extracellular signal-regulated kinase (ERK) and protein kinase C.

The discrepancies concerning LO supplementation effects on pro/antioxidant status markers among studies could be due to differences in the study design, such as rat models, supplementation duration, and LO formulations and dosages. Moreover, the specific mechanisms underlying the antioxidant action of LO have not been thbroughlly investigated yet. 

A growing body of evidences shows that HIIT is a time-efficient exercise mode for losing total and (intra)abdominal FM [8,58,59] and for reducing obesity-induced OS [20,28,60,61]. As already mentioned by [30], the 12-week HIIT program, but not LO supplementation, changed body composition (by limiting weight and FM gain and by decreasing adipocyte size in the mesenteric and subcutaneous adipose tissues only). Moreover, the HIIT program alleviated HFD negative effects in liver by reducing SOD activity, and also by decreasing the activity of the pro-oxidant enzyme NOx. In animals, very few studies have tested the effects of a HIIT program on obesity-induced OS [20,58,60,61]. Among those using HFD to induce obesity [58,60,61], there is no consensus on HIIT effect on the pro/antioxidant status because the HFD-induced OS alterations differed among studies. However, in all studies, including ours, HIIT corrected most of such alterations. In healthy people, moderate training [62] and more recently high intensity training [63,64] upregulate the activity of antioxidant enzymes through redox activation of NRF2 signaling. In our study and in others [65], liver SOD activity was increased by the obesity state, probably through ROS overproduction (especially its substrate, superoxide anion) via several mechanisms described by Vincent et al. [66]. We could hypothesize that unlike in healthy subjects, HIIT did not activate SOD (which was already activated by obesity), but lowered and normalized its activity by decreasing the activity of the various pathways implicated in obesity-induced ROS production (thus reducing mitochondrial ROS production) and also by lowering inflammation that promotes OS. We also found that HIIT decreased hepatic NOx activity, which was not increased by HFD. Here again, to our knowledge, we are the first to show a positive effect of HIIT on NOx in liver in a model of obesity. Our result is consistent with Alves et al. [67] who reported that cardiac NOx expression is increased in fructose diet-fed mice and that HIIT decreases this response. 

Finally, we hypothesized that LO supplementation and HIIT could potentiate their effects by normalizing the redox status in HFD-fed Wistar rats. The effects of the two combined interventions persisted (HIIT decreased liver SOD and NOx activities; LO decreased NOx activity), except for the increase in CAT activity induced by LO supplementation that disappeared when combined with training. The two interventions potentiated only SOD activity in subcutaneous fat. Indeed, on their own, LO and HIIT could not correct the HFD-induced decrease of SOD activity. Conversely, when combined, they increased SOD to a higher level than in control rats (LFD). Studies on the combined effects of antioxidant-based nutritional supplements and exercise training on the redox status are not unequivocal. In both healthy and pathological rodent models, some studies found beneficial and synergistic effects [68], but not others [69]. Moreover, some studies demonstrated that their combination attenuates and blunts their individual actions [70]. More molecular studies at the transcriptional level combining antioxidant supplementation and training are necessary to elucidate these protective mechanisms.

Concerning the main strengths of our study, this is the first work to assess the effects of the LO supplementation and HIIT combination. The potentiation of their effects on SOD is a key finding because SOD is a major antioxidant defense enzyme. This was also the first study to evaluate their effects on NOx, an important prooxidant enzyme that is upregulated in many tissue in obesity [71]. The reduction of its activity by the two treatments is important because it limits the production of superoxide anion that increases OS. In obesity, inflammation and OS are two self-perpetuating phenomena. The decrease in NOx activity by HIIT or LO supplementation helps to break this vicious circle [72].

Concerning the limitations, we did not study the expression and content of pro/antioxidant enzymes and the possible mechanism of action of the different treatments (signaling pathway activated by training for example). For practical reasons, training was not individualized in relation to the maximal aerobic speed of each rat. Although the individual intensity could have been theoretically different, leading to an intra-group variability in exercise intensity, the large number of rats (*n* = 17) included in each group should have limited this risk. Finally, it would have been interesting to monitor also the changes of these markers during the induction phase of obesity.

## 5. Conclusions

HIIT and LO supplementation had beneficial tissue-specific effects on the pro/antioxidant balance of obese rats. Although these strategies moderately corrected HFD deleterious effects in plasma, they exerted positive effects particularly in liver. Finally, the HIIT and LO supplementation combination potentiated SOD activity in subcutaneous adipose tissue. In the clinical practice, these two strategies for managing the redox status of obese patients can be proposed separately, but when used in combination, they might allow a synergistic action on SOD, one of the main antioxidant enzymes. In addition, our results suggest beneficial effects on the prooxidant/antioxidant balance. As ROS are involved in the development of cardiovascular diseases usually associated with obesity (e.g., atherosclerosis or hypertension), mainly via the activation of the prooxidant enzymes NOx and XO [73], the combination of LO supplementation and HIIT might be useful in such conditions. Nevertheless, studies in specific animal models and then in patients are needed to confirm this hypothesis.

## Figures and Tables

**Table 1:** Pro/antioxidant markers in the LFD (*n* = 12) and HFD (*n* = 12) groups at the end of phase 2 (training and/or LO supplementation for 12 weeks).

	LFD	HFD	*p*
Plasma			
GPx (μmol·min^−1^·g^−1^)	0.22 ± 0.02	0.21 ± 0.02	0.08
Liver			
AOPP (μmol·g^−1^)	3.40 ± 1.23	3.24 ± 1.06	0.73
MDA (μmol·g^−1^)	0.21 ± 0.16	0.29 ± 0.21	0.31
XO (μmol·min^−1^·g^−1^)	0.14 ± 0.05	0.18 ± 0.05	0.07
NOx (μmol·min^−1^·g^−1^)	0.72 ± 0.39	0.83 ± 0.24	0.44
CAT (μmol·min^−1^·g^−1^)	0.20 ± 0.03	0.19 ± 0.01	0.89
GPx (μmol·min^−1^·g^−1^)	3.58 ± 1.68	2.94 ± 1.34	0.31
Muscle			
AOPP (μmol·g^−1^)	9.51 ± 3.64	10.76 ± 8.01	0.96
MDA (μmol·g^−1^)	0.92 ± 0.22	0.9 ± 0.23	0.80
XO (μmol·min^−1^·g^−1^)	11.46 ± 2.13	11.85 ± 3.02	0.73
NOx (μmol·min^−1^·g^−1^)	10.99 ± 3.03	9.38 ± 2.64	0.19
SOD (μmol·min^−1^·g^−1^)	3.85 ± 1.61	3.18 ± 1.90	0.38
CAT (μmol·min^−1^·g^−1^)	1.96 ± 0.43	2.10 ± 0.95	0.65
GPx (μmol·min^−1^·g^−1^)	109.57 ± 24.96	118.96 ± 43.00	0.54
Subcutaneous adipose tissue			
AOPP (μmol·mg^−1^)	2.13 ± 1.30	1.83 ± 0.87	0.68
MDA (μmol·g^−1^)	4.42 ± 1.73	3.74 ± 1.79	0.35
XO (μmol·min^−1^·g^−1^)	2.27 ± 0.70	1.99 ± 0.40	0.24
NOx (μmol·min^−1^·g^−1^)	3.07 ± 0.70	2.67 ± 0.55	0.13
CAT (μmol·min^−1^·g^−1^)	1.83 ± 0.60	1.70 ± 0.39	0.55
GPx (μmol·min^−1^·g^−1^)	60.51 ± 22.90	49.29 ± 8.0	0.36
Epididymal adipose tissue			
AOPP (μmol·g^−1^)	25.11 ± 38.97	15.53 ± 11.84	0.77
MDA (μmol·g^−1^)	64.21 ± 67.60	26.47 ± 12.65	0.07
XO (μmol·min^−1^·g^−1^)	22.24 ± 9.56	23.57 ± 8.80	0.73
NOx (μmol·min^−1^·g^−1^)	22.54 ± 8.77	22.30 ± 7.52	0.85
CAT (μmol·min^−1^·g^−1^)	22.40 ± 16.38	12.95 ± 5.45	0.08
GPx (μmol·min^−1^·g^−1^)	119.56 ± 60.54	115.93 ± 57.36	0.88

**Table 2:** Pro/antioxidant markers in the HFD (*n* = 12), LO (*n* = 11), HIIT (*n* = 8), and HIIT + LO (*n* = 9) groups at the end of phase 2 (training and/or LO supplementation for 12 weeks). ^a,b^ Newman Keuls Post hoc test: means with the same letter indicate no significant difference. Any difference between two means carrying different.

	HFD (*n* = 12)	LO (*n* = 11)	HIIT (*n* = 8)	LO + HIIT (*n* = 9)	*p*
Liver					
AOPP (μmol·g^−1^)	3.24 ± 1.06	3. 40 ± 1.02	3.25 ± 1.40	2.98 ± 0.53	0.89
MDA (μmol·g^−1^)	0.29 ± 0.21	0.25 ± 0.20	0.21 ± 0.17	0.23 ± 0.19	0.23
XO (μmol·min^−1^·g^−1^)	0.17 ± 0.05 ^ab^	0.21 ± 0.05 ^b^	0.14 ± 0.03 ^a^	0.15 ± 0.04 ^a^	0.007
GPx (μmol·min^−1^·g^−1^)	2.95 ± 1.34	3.17 ± 0.87	2.36 ± 0.79	2.41 ± 0.78	0.15
Plasma					
AOPP (μmol·g^−1^)	0.07 ± 0.03	0.08 ± 0.04	0.08 ± 0.03	0.07 ± 0.02	0.82
CAT (μmol·min^−1^·g^−1^)	3.89 ± 0.70	4.07 ± 0.98	3.94 ± 0.68	3.56 ± 0.59	0.53
GPx (μmol·min^−1^·g^−1^)	0.21 ± 0.02	0.22 ± 0.02	0.22 ± 0.02	0.21 ± 0.02	0.74
Muscle					
AOPP (μmol·g^−1^)	10.76 ± 8.01	9.10 ± 3.34	11.48 ± 7.31	10.48 ± 5.71	0.94
MDA (μmol·g^−1^)	0.90 ± 0.23	0.90 ± 0.27	0.82 ± 0.33	1.00 ± 0.52	0.74
XO (μmol·min^−1^·g^−1^)	11.85 ± 3.02	11.85 ± 3.02	11.64 ± 4.22	10.92 ± 2.07	0.91
NOx (μmol·min^−1^·g^−1^)	9.38 ± 2.64	10.52 ± 3.46	11.21 ± 2.52	10.98 ± 2.09	0.48
SOD (μmol·min^−1^·g^−1^)	3.18 ± 1.90 ^ab^	2.02 ± 0.92 ^b^	4.14 ± 1.20 ^a^	3.52 ± 0.875 ^ab^	0.01
CAT (μmol·min^−1^·g^−1^)	2.10 ± 0.95	2.22 ± 0.86	2.63 ± 0.89	2.11 ± 0.71	0.55
GPx (μmol·min^−1^·g^−1^)	118.96 ± 43.00	115.81 ± 31.60	117.83 ± 40.40	100.28 ± 33.51	0.71
Subcutaneous adipose tissue					
AOPP (μmol·mg^−1^)	1.83 ± 0.87	1.64 ± 0.55	1.91 ± 1.03	1.52 ± 0.35	0.98
MDA (μmol·g^−1^)	3.74 ± 1.79 ^a^	3.35 ± 2.16 ^a^	6.46 ± 3.69 ^a^	5.95 ± 3.15 ^a^	0.03
XO (μmol·min^−1^·g^−1^)	1.99 ± 0.40	1.79 ± 0.78	1.95 ± 0.49	1.83 ± 0.79	0.87
NOx (μmol·min^−1^·g^−1^)	2.67 ± 0.55	2.33 ± 0.91	2.82 ± 0.64	2.46 ± 0.90	0.52
CAT (μmol·min^−1^·g^−1^)	1.70 ± 0.39	1.52 ± 0.51	1.47 ± 0.28	1.52 ± 0.26	0.55
Epididymal adipose tissue					
AOPP (μmol·g^−1^)	15.53 ± 11.84	15.64 ± 8.59	20.80 ± 9.40	16.41 ± 8.19	0.58
MDA (μmol·g^−1^)	26.47 ± 12.65	29.07 ± 20.02	29.75 ± 14.10	30.58 ± 15.44	0.94
XO (μmol·min^−1^·g^−1^)	23.57 ± 8.80	25.75 ± 8.69	28.16 ± 7.48	28.63 ± 15.73	0.69
NOx (μmol·min^−1^·g^−1^)	22.30 ± 7.52	23.74 ± 8.16	25.39 ± 6.48	29.74 ± 14.44	0.44
CAT (μmol·min^−1^·g^−1^)	12.95 ± 5.45	15.71 ± 10.98	16.36 ± 8.10	19.00 ± 14.09	0.61

## Supplementary Materials

The following are available online at https://www.mdpi.com/article/10.3390/nu13103531/s1, Table S1: Diet composition.

## Abbreviations

AOPP: advanced oxidation protein products, CAT: catalase, GPx: glutathione peroxidase, HFD: high-fat diet, HIIT: high-intensity interval training, LFD: low-fat diet, LO: linseed oil, NOx: NADPH oxidase, oxLDL: oxidized low density lipoprotein, SOD: superoxide dismutase, XO: xanthine oxidase.
