# Supplementary material for: Beneficial Effects of High Intensity Interval Training and/or Linseed Oil Supplementation to Limit Obesity-Induced Oxidative Stress in High Fat Diet-Fed Rats
Source: Nutrients. 2021 Oct 9;13(10):3531. doi: 10.3390/nu13103531 (PMC8537033; doi:10.3390/nu13103531)
Supplement: Supplementary file 1 [file nutrients-13-03531-s001.zip › nutrients-1402714-supplementary.pdf]

Supplementary Table S1: Diet composition

Ingredient Amount (mg/kg)

AIN-93-VX Vitamin Mix:
Nicotinic Acid 3.00
D-Calcium Pantothenate 1.60
Pyridoxine HCl 0.70
Thiamine HCl 0.60
Riboflavin 0.60
Folic Acid 0.20
D-Biotin 0.02
Vitamin B12 (0.1% triturated in mannitol) 2.50
α-Tocopherol Powder (250 U/gm) 30.00
Vitamin A Palmitate (250,000 U/gm) 1.60
Vitamin D3 (400,000 U/gm) 0.25
Phylloquinone 0.075
Powdered Sucrose 959.655

AIN-93G Mineral Mix
Calcium Carbonate 35.7%
Monopotassium phosphate 19.6%
Potassium Citrate monohydrate 7.078%
Sodium Chloride 7.4%
Potassium Sulfate 4.66%
Magnesium Oxide 2.4%
Ferric Citrate 0.606%
Zinc Carbonate 0.165%
Manganese Carbonate 0.063%
Copper Carbonate 0.03%
Potassium Iodate 0.001%
Sodium Selenate, Anhydrous 0.00103%
Ammonium Molybdate.4H2O 0.000795%
Sodium Metasilicate.9H2O 0.145%
Chromium Potassium Sulfate.12H2O 0.0275%
Lithium Chloride 0.00174%
Boric Acid 0.008145%
Sodium Fluoride 0.00635%
Nickel Carbonate 0.00318%
Ammonium Vanadate 0.00066%
Powdered Sugar 22.1%
